# Genetic relatedness among indigenous rice varieties in the Eastern Himalayan region based on nucleotide sequences of the *Waxy* gene

**DOI:** 10.1186/1756-0500-7-953

**Published:** 2014-12-29

**Authors:** Baharul I Choudhury, Mohammed L Khan, Selvadurai Dayanandan

**Affiliations:** Biology Department, Forest and Evolutionary Genomics Laboratory, and Centre for Structural and Functional Genomics, Concordia University, 7141 Sherbrooke St. West, Montreal, Quebec H4B 1R6 Canada; Québec Centre for Biodiversity Sciences, Montréal, QC Canada; Department of Botany, Dr. Harisingh Gour Central University, Sagar, MP India

**Keywords:** *Boro*, Ecotype, Genetic relatedness, Indigenous rice varieties, *Jum*, *Sali*

## Abstract

**Background:**

Indigenous rice varieties in the Eastern Himalayan region of Northeast India are traditionally classified into *sali*, *boro* and *jum* ecotypes based on geographical locality and the season of cultivation. In this study, we used DNA sequence data from the *Waxy* (*Wx*) gene to infer the genetic relatedness among indigenous rice varieties in Northeast India and to assess the genetic distinctiveness of ecotypes.

**Findings:**

The results of all three analyses (Bayesian, Maximum Parsimony and Neighbor Joining) were congruent and revealed two genetically distinct clusters of rice varieties in the region. The large group comprised several varieties of *sali* and *boro* ecotypes, and all agronomically improved varieties. The small group consisted of only traditionally cultivated indigenous rice varieties, which included one *boro*, few *sali* and all *jum* varieties. The fixation index analysis revealed a very low level of differentiation between *sali* and *boro* (*F*_ST_ = 0.005), moderate differentiation between *sali* and *jum* (*F*_ST_ = 0.108) and high differentiation between *jum* and *boro* (*F*_ST_ = 0.230) ecotypes.

**Conclusion:**

The genetic relatedness analyses revealed that *sali*, *boro* and *jum* ecotypes are genetically heterogeneous, and the current classification based on cultivation type is not congruent with the genetic background of rice varieties. Indigenous rice varieties chosen from genetically distinct clusters could be used in breeding programs to improve genetic gain through heterosis, while maintaining high genetic diversity.

**Electronic supplementary material:**

The online version of this article (doi:10.1186/1756-0500-7-953) contains supplementary material, which is available to authorized users.

## Background

**Figure 1:**
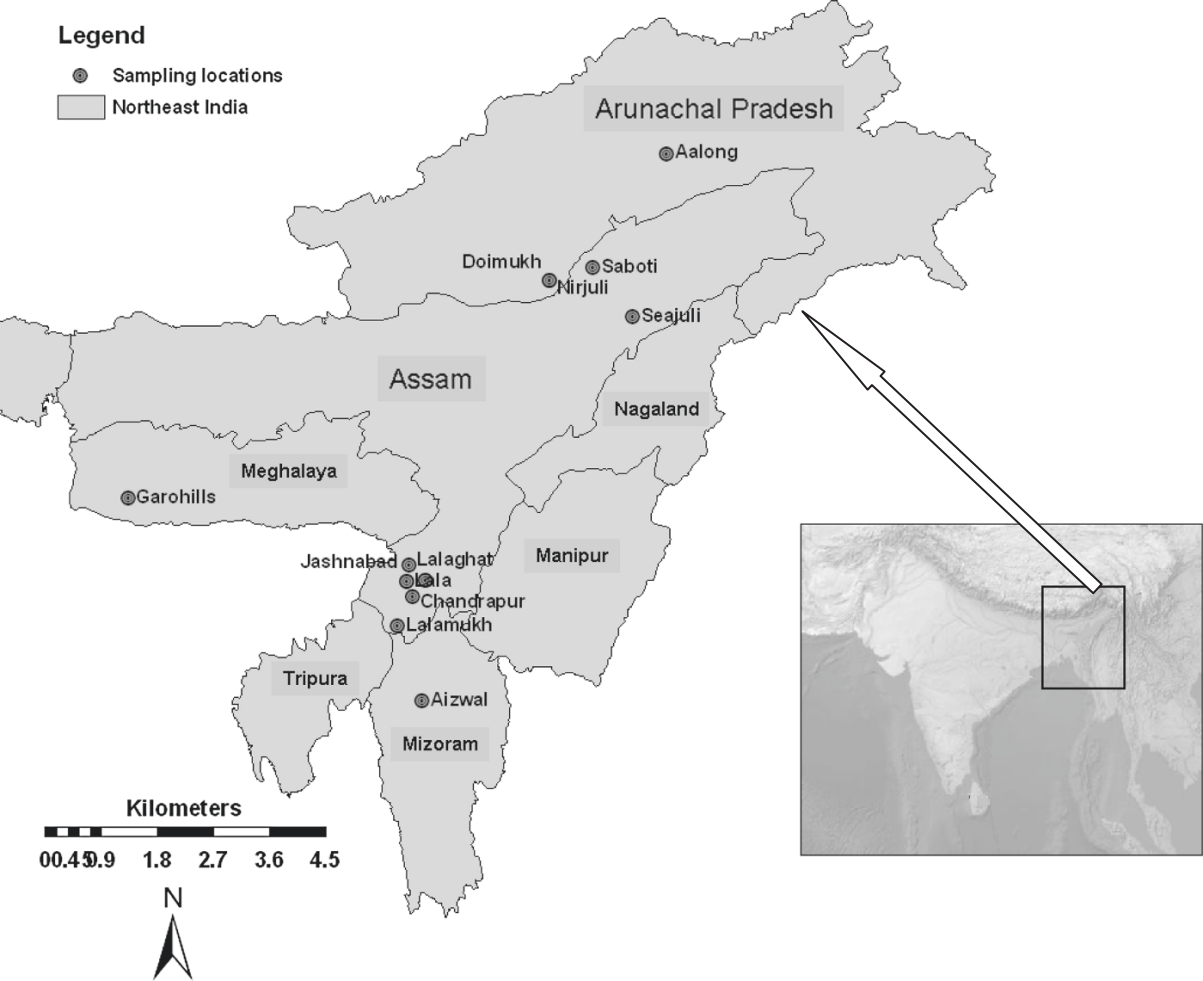
**Map of NE India showing sampling sites of indigenous rice varieties.**

The Eastern Himalayan region of Northeast (NE) India, which spans over 255,000 km^2^ covering Arunachal Pradesh, Assam, Manipur, Meghalaya, Mizoram, Nagaland and Tripura states (Figure 1) is home to a large number of indigenous rice varieties [1–3]. Such varieties are cultivated under diverse agro-climatic conditions and distributed over a broad geographical area ranging from flood plains and lower catchment areas of the Brahmaputra and Barak rivers to high altitude mountains of the Himalayas. Based upon habitat type and season of cultivation, these rice varieties are classified into three ecotypes: *sali*, *boro* and *jum*. The *sali* and *boro* ecotypes are cultivated in irrigated lands in low-lying areas, whereas varieties of the *jum* ecotype are cultivated in dry upland areas. The varieties of *sali* ecotype are cultivated during the warm and wet summer months (June through December), and the *boro* ecotype is cultivated during the cold and dry winter months (November through May). Cultivation of the *boro* ecotype in NE India has recently increased due to the improvement of irrigation infrastructure in the region. The *jum* varieties are cultivated during the rainy season (March through November) in upland shifting cultivation lands known as *jum* agricultural systems practiced by local tribal communities in the hilly areas of NE India [4].

The indigenous rice varieties in NE India show remarkable diversity in morphological and agronomic traits including high variability in size, shape, aroma and nutritional properties of grains [5], disease resistance [6] and abiotic stress tolerance [7]. A recent study revealed high levels of genetic diversity in these rice varieties with the highest genetic diversity in the varieties of the *sali* ecotype, followed by the *jum* and *boro* ecotypes [2]. These rice varieties with exceptional phenotypic and genetic diversity can serve as an important source of germplasm for the genetic improvement of cultivated rice. A thorough understanding of genetic relatedness among these rice varieties is crucial for designing breeding programs for the genetic improvement of rice, allowing us to capitalize on genetic gain through heterosis while maintaining high genetic diversity.

The objective of the present study is to infer the genetic relatedness among indigenous rice varieties of *sali*, *boro* and *jum* ecotypes cultivated in NE India using the nucleotide sequences of the *Wx* gene. As a single copy nuclear gene with high polymorphism, the nucleotide sequences of the *Wx* gene is an ideal genomic tool to assess the genetic relatedness of rice varieties. The *Wx* gene, which encodes granule-bound starch synthase [8, 9], determines the amylose content in the endosperm and influences the glutinous nature of the rice grain. The nucleotide sequences of three *Wx* genes (*Wx-A1*, *Wx-B1* and *Wx-*D1) reported from wheat [10] have been used successfully to infer genetic relatedness among wheat cultivars [11], highlighting the *Wx* gene’s suitability for determining genetic relatedness in crop plants.

## Methods

### Plant samples

A total of 29 samples (Table 1) were collected from NE India for this study, including 21 *sali* (5 of which were agronomically improved varieties), 4 *jum* and 4 *boro*. Either seeds or fresh leaf samples were collected from the field, and the data on ecotype, morphology and grain characteristics were gathered through direct observation and/or interviewing farmers. Seeds were germinated in petri dishes before being transferred into pots and seedlings were grown in the green house. Leaf samples from seedlings were harvested and air-dried before use. The total genomic DNA from dry leaves was extracted following modified cetyltrimethyl ammonium bromide (CTAB) DNA extraction protocol [12, 13]. *Oryza rufipogon* was used as an outgroup in all analyses.

**Table 1:** **The variety name, cultivation type and collection sites of traditionally cultivated indigenous and agronomically improved rice varieties in Northeast India (AP, Arunachal Pradesh; AS, Assam; ML, Meghalaya; MZ, Mizoram)**

Variety Name	Type	Location
Bas Beroin	Sali	Cachar (AS)
Balam	Sali	Cachar (AS)
Lahi	Sali	Doimukh (AP)
Local Basmati	Sali	Doimukh (AP)
Joha	Sali	Doimukh (AP)
Lallatoi	Sali	Hailakandi (AS)
Arfa	Sali	Hailakandi (AS)
Mulahail	Sali	Hailakandi (AS)
Guaroi	Sali	Hailakandi (AS)
Harinarayan	Sali	Hailakandi (AS)
Bherapawa	Sali	Hailakandi (AS)
Kakiberoin	Sali	Hailakandi (AS)
Borjahinga	Sali	N. Lakhimpur, (AS)
Til Bora	Sali	N. Lakhimpur, (AS)
Hati Hali	Sali	N. Lakhimpur, (AS)
Ranga Borah	Sali	N. Lakhimpur, (AS)
Ranjit	Sali (Improved)	Hailakandi (AS)
IR8	Sali (Improved)	Hailakandi (AS)
Bahadur	Sali (Improved)	Hailakandi (AS)
Pankaj	Sali (Improved)	Hailakandi (AS)
Joya	Sali (Improved)	Hailakandi (AS)
Kawanglawang	Jum	Aizwal, (MZ)
Sorpuma	Jum	Doimukh (AP)
Mimutim	Jum	Garo Hills (ML)
Papue	Jum	West Siang (AP)
Borua Beroin	Boro	Cachar (AS)
Aubalam	Boro	Cachar (AS)
Bashful	Boro	Cachar (AS)
Moircha	Boro	Cachar (AS)
*O. rufipogon*	Wild	Eastern India

### PCR amplification and sequencing

A selected region of the *Wx* gene (~2.7 kb), which included the promoter, exon 1, intron 1, the 5′ end of exon 2, and the entire non-coding region within exon 2, was amplified using several oligonucleotides (Table 2) as described in Olsen and Purugganan [14]. PCR amplifications were performed in an Applied Biosystems thermal cycler in a total volume of 25 μL reaction mixture consisting of 0.25 mM dNTP, 2.0 mM MgCl_2_, 2.5 μL of 10X buffer, 1.5 pmol of each primer and 0.2 U *Taq* polymerase. For the PCR amplification with primer pairs WxU1F-Wx1R and Wx2Fa-Wx2R, we used a touchdown thermal cycling profile with initial denaturation at 94°C for 2 minutes followed by denaturation at 94°C for 30 seconds, annealing at 70°C for 30 seconds and extension at 72°C for 2 minutes. The annealing temperature was lowered at a rate of 1°C per cycle starting at 70°C and reaching to 65°C. Additional 30 cycles of thermocycling were performed with annealing at 65°C for 30 seconds, extension at 72°C for 2 minutes, and denaturation at 94°C for 30 seconds, and followed with a final extension at 72°C for 5 min. The thermocycling profile used for PCR amplification with the primer pair WxU1Fint- Wx2Rint included initial denaturation at 94° for 2 min followed by 35 cycles of 94° for 30 sec, 55° for 30 sec, 72° for 2 min and a final extension of 72° for 5 min. The amplified DNA fragments were separated through electrophoresis on 1% agarose gels containing 0.33 μg/ml ethidium bromide and the size of the amplification product was determined using GeneRuler 1 kb DNA ladder (Fermentas) as a size standard (Additional file 1: Figure S1). The PCR products were either directly sequenced or sequenced after purification using Bio-Basic PCR product purification kit (Bio-Basic Inc.). The DNA sequencing was performed in Applied Biosystems 3730 × l DNA analyzer at the Genome Québec Innovation Centre at McGill University.

**Table 2:** **Oligonucleotide primer sequences used for amplification of the** ***Wx*** **gene**

Forward primer	Reverse primer
WxU1F5′-GCCGAGGGACCTAATCTGC-3′	Wx1R5′-TGGTGTGGGTGGCTATTTGTAG-3′
Wx2Fa5′-GCCCCGCATGTCATCGTC-3′	Wx2R5′-GTTGTCTAGCTGTTGCTGTGGA-3′
WxU1Fint5′-TTGTCAGCACGTACAAGCA-3′	Wx2Rint5′-GCTATATACATTTTCCTTTGACCAA-3′

### Data analysis

The DNA sequences were analyzed using the computer program Geneious version 5.4.6 (http://www.geneious.com/). The resulting consensus sequences were aligned using the software program ClustalW v2 [15]. We used Bayesian, maximum-parsimony (MP) and neighbor-joining (NJ) methods to infer genetic relatedness of rice varieties. The Bayesian analysis infers the phylogenetic relationships based on posterior probability distribution using evolutionary models [16], whereas the MP analysis infers the evolutionary tree(s) with the minimum number of nucleotide changes [17]. The NJ method uses a pairwise distance matrix to infer the genetic relatedness among taxa [18]. Thus, the use of a variety of approaches that differ in underlying assumptions provided a means to assess the robustness of resulting phylogenetic trees.

The Bayesian analysis was performed using the computer program MrBayes v3.2.1 [19]. The posterior probabilities were estimated by sampling trees using the Markov Chain Monte Carlo (MCMC) method [20]. The parameters for prior probability distributions were set as follows: rates = invgamma (gamma-shaped rate variation with a proportion of invariable sites); statefreqpr = Dirchlet (100,100,100,100) (Dirichlet distributions as prior with more emphasis on equal nucleotide frequencies). The nucleotide sequence matrix was analyzed using Modeltest [21] to determine the most suitable model of nucleotide substitution. The results of the Modeltest analysis revealed that the HKY + I + G model (Hasegawa, Kishino, Yano model with a proportion of invariable sites plus gamma distributed rate variation) [22] as the best model for the *Wx* gene sequence data set (Table 3). The MCMC sampling was performed for four chains and run for 1,000,000 generations. The tree sampling was done at every 100 generations with burn-in fraction set at 0.25 (burninfrac = 0.25) to discard the first 25% trees from the cold chain. Five independent runs were performed and the consensus phylogram of the resulting trees was viewed in FigTree v1.3.1 [23].

**Table 3:** **The best model of nucleotide substitution obtained through Modeltest analy** **ses based on Akaike Information Criterion (AIC)**

Model selected	HKY + I + G
-lnL	4690.042
AIC	9392.084
Base frequencies	A = 0.275
	C = 0.239
	G = 0.213
	T = 0.273
Substitution model	Ti/Tv ratio = 3.489
Among-site rate variation	Proportion of invariable sites (I) = 0.937
Variable sites (G)	Gamma distribution shape parameter = 0.148
Using mixed χ^2^ distribution	P-value = < 0.00001

The phylogenetic trees based on NJ and MP methods were inferred using the PAUP* [24] software. Kimura 2-parameter distances [25] were used in the NJ analysis following Saitou and Nei [18]. The MP analyses were performed with full heuristic search with tree bisection-reconnection branch swapping and random order of taxon addition option. The robustness of tree topologies was tested with 1000 bootstrap replicates. Nodes with greater than 50% bootstrap support were retained in the tree.

Genetic relatedness among rice varieties was further analyzed through haplotype networks. In this analysis, a series of nested clades based on haplotypic or allelic networks were reconstructed. The haplotype network analysis infers evolutionary relationships among intraspecific populations and closely related species [26]. The median-joining algorithm [27] as implemented in the software package NETWORK 4.5.1 (Fluxus Technology) was used in this analysis. The level of differentiation between the ecotypes was estimated by calculating *F*_ST_ values between pairs of populations using the DnaSP software [28].

## Results

**Figure 2:**
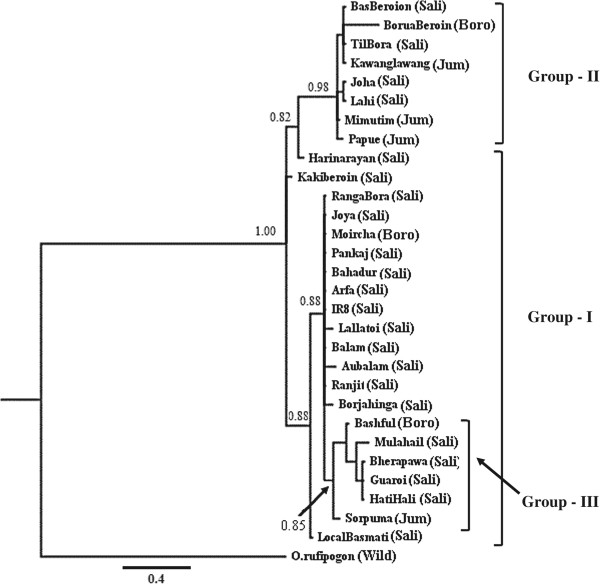
**Bayesian phylogram of rice ecotypes based on the nucleotide sequences of the** ***Wx*** **gene.** Abbreviations in brackets represent ecotype and numbers over the nodes represent posterior probability values. Group-III is the subgroup within Group-I.

The length of the aligned sequence matrix of the *Wx* gene was 2770 nucleotides and contained 7 microsatellite alleles at the 5′ untranslated region of exon 1. Altogether, 84 SNPs (on average 1 SNP for 32.98 bp) were detected. The Bayesian, MP and NJ clustering methods resulted in two major clades with similar tree topologies with high statistical support. In the Bayesian tree (Figure 2), the major clade (Group-I) comprised both indigenous and agronomically improved rice varieties. The majority of varieties in this group were of *sali* ecotype. The other clade (Group-II) consisted of only indigenous varieties with predominant representation of *jum* and *sali* ecotype varieties and one variety of the *boro* ecotype. Within Group-I, a small subgroup (Group-III) comprising six indigenous varieties representing all three ecotypes was found. Two *sali* varieties (*Local Basmati* and *Harinarayan*) were basal to Group-I and Group-II respectively.

The MP analysis resulted in 141 equally parsimonious trees with total length of 140 steps, and the consensus tree topology was similar to the tree based on the Bayesian analysis. Most varieties of the *sali* ecotype clustered within Group-I along with the varieties of *boro* and *jum* ecotypes (Additional file 1: Figure S2). The other clade (Group-II) comprised only indigenous rice varieties. The varieties clustered within Group-III were identical to the group that clustered together in the Bayesian analysis. The sole difference between the Bayesian and MP-based trees was the placement of two varieties of the *sali* ecotype (*Harinarayan* and *Kakiberoin*), which occupied a basal position in Group-II in the former analysis and in Group-I in the latter. The NJ analysis also showed similar tree topology, except for the Group-III varieties, which formed a separate cluster and occupied a basal position in Group -I (Additional file 1: Figure S3).

**Figure 3:**
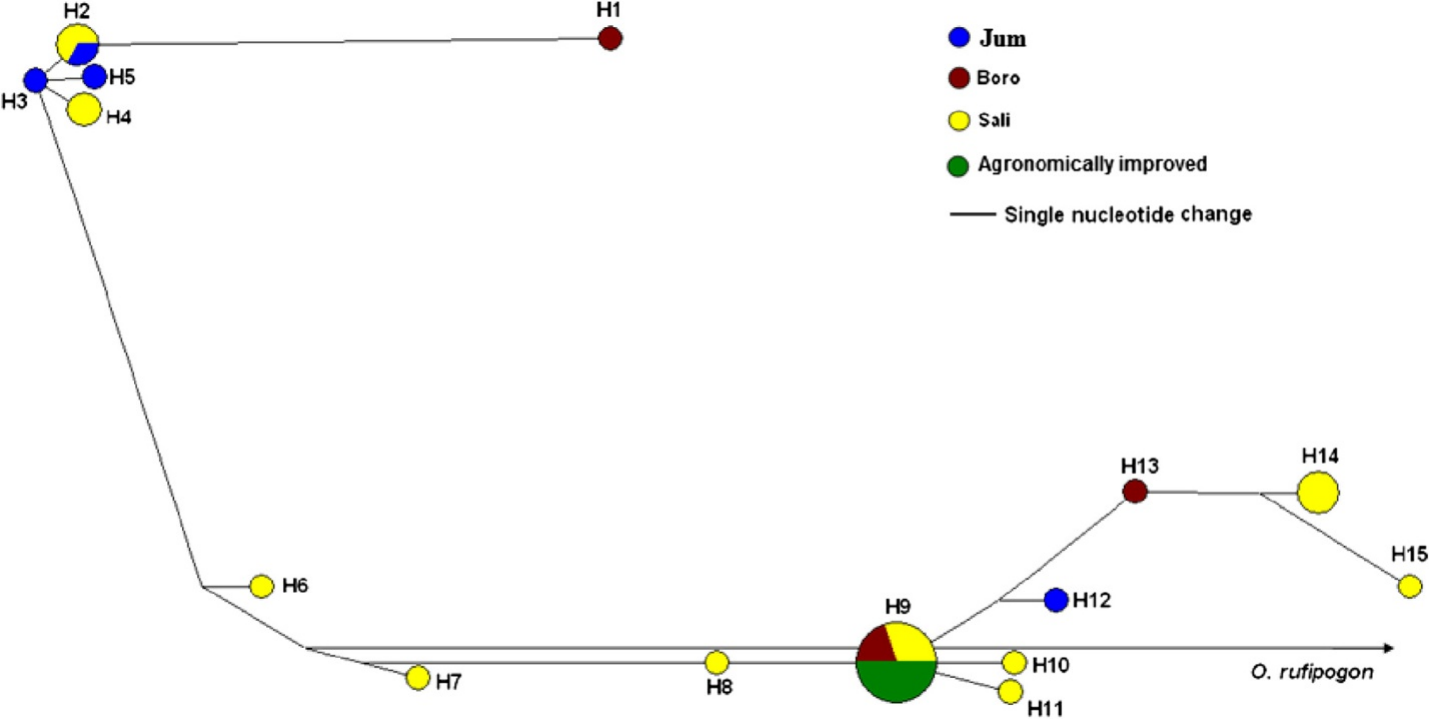
**Haplotype networks for the** ***Wx*** **gene.** Each node represents a haplotype and size of the circle is proportional to its frequency.

A total of 109 substitution polymorphisms grouped into 16 distinct haplotypes were detected in the *Wx* nucleotide sequence matrix (Figure 3; Additional file 2: Table S1). The *Wx* haplotype network formed two main groups comprising haplotypes 1–5 in one group and haplotypes 6–15 in the other group. The larger haplotype group (H8) consisted of 10 varieties representing two ecotypes and all agronomically improved varieties. A few haplotypes, mostly varieties of the *sali* ecotype differed at one to four substitutions and grouped together with the larger haplotype group. The other haplotype groups (H1 – H5) consisted exclusively of indigenous varieties representing at least one variety from each of the three ecotypes. Population differentiation analysis showed very low to moderate levels of differentiation among different ecotypes (Table 4). The lowest *F*_ST_ value was detected between *sali* and *boro* (0.005) and the highest between *jum* and *boro* (0.230) ecotypes.

**Table 4:** **The pairwise differentiation (** ***F***_**ST**_ **) of ecotypes**

Ecotypes	***Wx***gene
*Sali* and *Boro*	0.005
*Sali* and *Jum*	0.108
*Jum* and *Boro*	0.230

## Discussion

In the present study, we investigated genetic relatedness among three different rice ecotypes in the eastern Himalyan region of NE India. The Bayesian, MP and haplotype network analyses resulted in similar tree topologies consisting of two major groups. This clustering pattern was not congruent with three commonly cultivated ecotypes (*sali*, *boro* and *jum*) in NE India, and suggests a polyphyletic nature of rice ecotypes [29]. This could be attributable to two possible reasons. First, exchange of seed material between regions mediated through human migration [30], often associated with migration of traditional farmers seeking better opportunities [31], could lead to cultivation of genetically different varieties within a given geographical locality. Second, large scale flooding during monsoon rainy seasons often damages crop plants, and farmers generally seek seeds from other regions leading to seed exchange between different agroclimatic regions. The polyphyletic nature of rice varieties in the region is in agreement with a previous study based on chloroplast DNA, which suggested polyphyletic maternal lineages for *O. sativa* ssp. *indica* [32]. Similar results were also reported in other crop species, including sweet sorghum and grain sorghum lines of *Sorghum bicolor* ssp. *bicolor* [33]. Based on the nucleotide sequences of the *Wx* gene, eight to ten genetically distinct indigenous rice varieties within Group-II are discernible. Similarly, rice varieties in the genetically distinct Group III may also contain unique genotypes. Thus, these indigenous rice varieties can serve as a valuable germplasm for genetic improvement of cultivated rice.

Cultivated rice has been subject to human mediated selection for various traits of agronomic and ecological importance. Adaptation to various agroclimatic conditions and human-mediated selection may have contributed to diversification of rice varieties in the NE Indian region [34]. The *jum* and *boro* ecotypes showed a high level of population differentiation (*F*_ST_ = 0.230), indicating local adaptation to contrasting habitats leading to high level of population differentiation [35–37]. The cultivation of varieties of the *jum* ecotype in dry, upland habitats, and the cultivation of varieties of the *boro* ecotype in low-lying irrigated land during the winter season may have led to the genetic isolation and genetic differentiation of varieties of these two ecotypes. Very low *F*_ST_ value (0.005) between *sali* and *boro* ecotypes at the *Wx* gene reflects high levels of gene flow between rice varieties of these two ecotypes [38] or the latter ecotype may have originated from the *sali* ecotype. Since cultivated rice is mostly self-pollinating [39], gene flow among varieties is minimal. Thus, the observed low differentiation between these two ecotypes could be attributable to the fact that the *boro* ecotype may have been selected from the *sali* ecotype to grow in low-lying areas during the winter season.

## Conclusion

The present study based on the nucleotide sequence data of the *Wx* gene revealed a) the polyphyletic nature of *sali*, *boro* and *jum* rice ecotypes and b) two genetically distinct groups of rice varieties in NE India. One group consisted of only traditionally cultivated varieties, while the other group comprised both agronomically improved and traditionally cultivated rice varieties. The occurrence of genetically distinct groups of rice varieties in the region highlights the importance of rice genetic resources in NE India as potential source of germplasm for genetic improvement of cultivated rice to maintain global food security under changing climatic conditions.

### Availability of supporting data

The aligned DNA sequences and phylogeny trees were submitted to TreeBASE (Accession number S14972) which can be accessed from the URL http://purl.org/phylo/treebase/phylows/study/TB2:S14972.

## Electronic supplementary material

Additional file 1: Figure S1: A sample agarose gel image of 22 rice samples showing PCR product using primer pairs WxU1Fint and Wx2Rint (see Table 2 for sequence details). **Figure S2.** The single most parsimonious tree based on maximum parsimony analysis identified through heuristic search of *Wx* nucleotide sequence data. Numbers above branches indicate branch lengths (number of nucleotide substitution) and bracketed numbers below indicate bootstrap values. **Figure S3.** The neighbor-joining tree based on nucleotide sequences of the *Wx* gene. Numbers above branches indicate branch length. (DOC 159 KB)

Additional file 2: Table S1: Polymorphic sites at the *Wx* locus of the 16 haplotypes detected in 29 cultivated rice (*O. sativa*) and *O. rufipogon* (W). Numbers in parentheses indicate the numbers of varieties per haplotype. (DOC 94 KB)
