# Supplementary material for: Genetic relatedness among indigenous rice varieties in the Eastern Himalayan region based on nucleotide sequences of the Waxy gene
Source: BMC Res Notes. 2014 Dec 29;7:953. doi: 10.1186/1756-0500-7-953 (PMC4320456; doi:10.1186/1756-0500-7-953)
Supplement: Supplementary file 1 — Additional file 1: Figure S1: A sample agarose gel image of 22 rice samples showing PCR product using primer pairs WxU1Fint and Wx2Rint (see Table 2 for sequence details). Figure S2. The single most parsimonious tree based on maximum parsimony analysis identified through heuristic search of Wx nucleotide sequence data. Numbers above branches indicate branch lengths (number of nucleotide substitution) and bracketed numbers below indicate bootstrap values. Figure S3. The neighbor-joining tree based on nucleotide sequences of the Wx gene. Numbers above branches indicate branch length. (DOC 159 KB) [file 13104_2013_3465_MOESM1_ESM.doc]

**
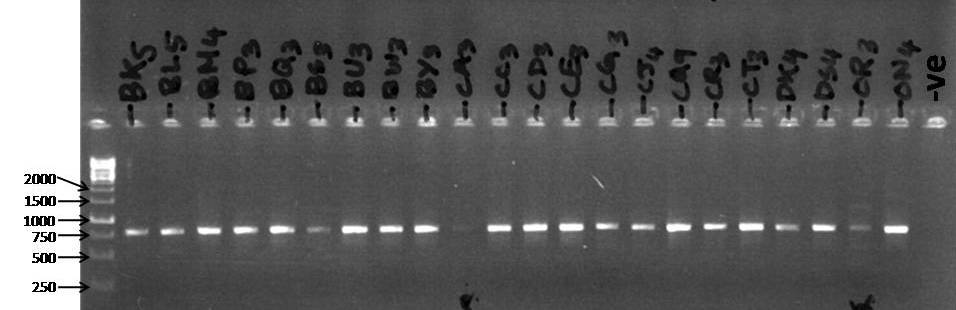
**

**Supplementary** **Figure S1**: A sample agarose gel image of 22 rice samples showing PCR product using primer pairs WxU1Fint and Wx2Rint (see Table 2 for sequence details).

**
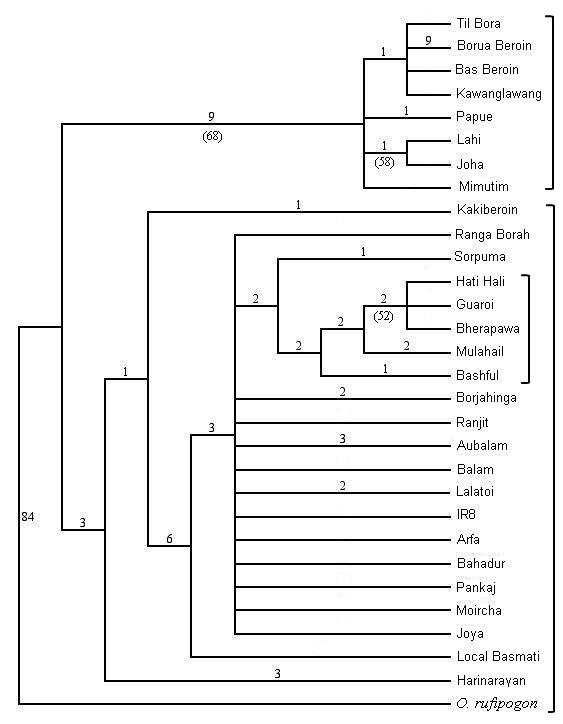
**

**Group - II**

**Group - III**

**Group - I**

**Supplementary** **Figure S2:** The single most parsimonious tree based on maximum parsimony analysis identified through heuristic search of *Wx* nucleotide sequence data. Numbers above branches indicate branch lengths (number of nucleotide substitution) and bracketed numbers below indicate bootstrap values.


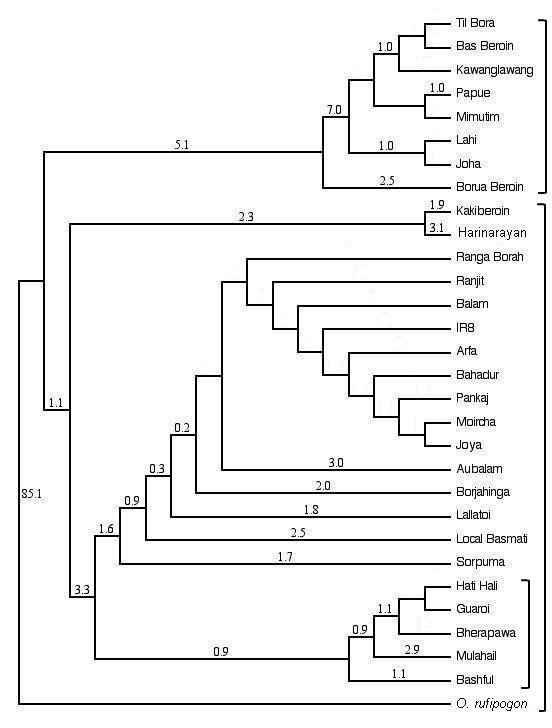


**Group - II**

**Group - I**

**Group - III**

**Supplementary** **Figure S3**: The neighbor-joining tree based on nucleotide sequences of the *Wx* gene. Numbers above branches
