# Supplementary material for: Genetic relatedness among indigenous rice varieties in the Eastern Himalayan region based on nucleotide sequences of the Waxy gene
Source: BMC Res Notes. 2014 Dec 29;7:953. doi: 10.1186/1756-0500-7-953 (PMC4320456; doi:10.1186/1756-0500-7-953)
Supplement: Supplementary file 2 — Additional file 2: Table S1: Polymorphic sites at the Wx locus of the 16 haplotypes detected in 29 cultivated rice (O. sativa) and O. rufipogon (W). Numbers in parentheses indicate the numbers of varieties per haplotype. (DOC 94 KB) [file 13104_2013_3465_MOESM2_ESM.doc]

**Supplementary Table 1:** Polymorphic sites at the *Wx* locus of the 16 haplotypes detected in 29 cultivated rice (*O. sativa*) and *O. rufipogon* (W). Numbers in parentheses indicate the numbers of varieties per haplotype.
